# Prevalence and Structure of HIV-1 Drug Resistance to Antiretrovirals in the Volga Federal District in 2008–2019

**DOI:** 10.3390/v14091898

**Published:** 2022-08-27

**Authors:** Olga Peksheva, Elena Kuzovatova, Olga Parfenova, Natalia Zaytseva

**Affiliations:** Academician I.N. Blokhina Nizhny Novgorod Scientific Research Institute of Epidemiology and Microbiology of the Federal Service for Surveillance on Consumer Rights Protection and Human Wellbeing, 71, Malaya Yamskaya St., 603950 Nizhny Novgorod, Russia

**Keywords:** HIV-1, drug resistance, antiretrovirals, prevalence, Sanger sequencing

## Abstract

The increasing number of HIV-infected people who are receiving ART, including those with low adherence, is causing the spread of HIV drug resistance (DR). A total of 1396 plasma samples obtained from treatment-experienced patients from the Volga federal district (VFD), Russia, were examined to investigate HIV DR occurrence. The time periods 2008–2015 and 2016–2019 were compared. Fragmentary Sanger sequencing was employed to identify HIV resistance to reverse transcriptase inhibitors (RTIs) and protease inhibitors (PIs) using an ABI 3500XL genetic analyzer, a ViroSeq™ HIV-1 genotyping system (Alameda, CA, USA) and AmpliSense HIV-Resist-Seq reagent kits (Moscow, Russia). In 2016–2019, HIV DR was detected significantly more often than in 2008–2015 (*p* < 0.01). Mutations to RTIs retained leading positions in the structure of DR. Frequencies of resistance mutations to nucleoside and non-nucleoside RTIs (NRTIs and NNRTIs) in the spectra of detected mutations show no significant differences. Resistance to NRTIs after 2016 began to be registered more often as a part of multidrug resistance (MDR), as opposed to resistance to a single class of antiretrovirals. The frequency of DR mutations to PIs was low, both before and after 2016 (7.9% and 6.1% in the spectrum, respectively, *p* > 0.05). MDR registration rate became significantly higher from 2008 to 2019 (17.1% to 72.7% of patients, respectively, *p* < 0.01). M184V was the dominant replacement in all the years of study. A significant increase in the frequency of K65R replacement was revealed. The prevalence of integrase strand transfer inhibitor (INSTI) resistance mutations remains to be investigated.

## 1. Introduction

Despite the emergence and spread of new infectious diseases in the world, HIV infection continues to be an important public health problem due to its continuing high mortality rate and the severity of its clinical course. In order to end the global AIDS epidemic, an increase in the population’s coverage with HIV testing and effective antiretroviral therapy (ART) is required.

In 2018, the Russian Federation for the first time reported national progress towards UNAIDS 90-90-90 goals: in 2017 81% of Russians living with HIV knew their status, 45% of them received treatment and 75% of those who received treatment achieved a suppressed viral load (VL) [1]. In 2021, ART coverage in Russia amounted to 82.2% of those who were under medical care/56.4% of those living with HIV. A total of 527,705 patients (that is, 79.9% of those on ART, or 46.4% of known Russians living with HIV) have achieved an undetectable VL [2].

The Volga federal district (VFD) has a high level of HIV prevalence and significant HIV incidence rate in the population. The average incidence rate in the VFD in 2021 was 49.6^0^/_0000_, which is 2.3% higher than the 2020 value (48.5^0^/_0000_) and 0.9% higher than the all-Russian indicator. An important factor in determining the dynamics of the epidemic process in the VFD is ART coverage of patients—on an average level in the VFD in 2021, 82.5% of patients who were under care received ART, which is twice as high as that in 2016 [3].

ART significantly reduces the incidence and mortality from HIV infection, improves the quality of patients’ lives, and minimizes the risk of further virus transmission [4]. However, its expansion may increase the risk of the virus developing resistance to HIV drugs [5].

The spread of mutant HIV strains that are resistant to antiretrovirals creates significant limitations during therapy, increases the risk of treatment ineffectiveness, and leads to an increase in transmissible drug resistance (TDR) in the population of HIV-infected ART naïve people [6].

Preventing and managing the emergence of HIV DR is a key component of a comprehensive and effective HIV response. In order to minimize the emergence and spread of HIV DR, the WHO recommends that antiretroviral therapy and pre-exposure prophylaxis programmes be accompanied by monitoring and surveillance of drug resistance [7].

Preventing the emergence of and combating DR will become increasingly important as the practice of early initiation of antiretrovirals for the treatment and prevention of HIV infection, as well as issues related to adherence, become widespread in the world [8,9,10,11,12].

Analysis of the prevalence of HIV DR mutations in the VFD allows, within the framework of epidemiological surveillance, development of approaches to prevent the occurrence and limit the spread of resistant HIV strains in the district.

Taken together, these factors support the need for continuing studies to determine the prevalence of HIV DR. In order to develop measures aimed at reducing the spread of HIV infection, it is important to know both the past and current molecular genetic characteristics of the epidemic [6].

The aim of this study is to determine the prevalence of mutations causing DR in response to the use of various classes of HIV drugs in HIV-infected patients from ten regions of the VFD during the period of 2008–2019.

## 2. Materials and Methods

From 2008 to 2019, 2018 samples of blood plasma of HIV-positive patients were delivered to the laboratory of the Privolzhsky Okrug Center for the Prevention and Control of AIDS of the Academician I.N. Blokhina Nizhny Novgorod Scientific Research Institute of Epidemiology and Microbiology of Rospotrebnadzor, for the purpose of identifying HIV DR mutations. Blood plasma samples were derived from patients from 10 areas of the VFD, including the republics of Mari El and Mordovia, the Udmurt Republic, the Chuvash Republic, the Kirov, Nizhny Novgorod, Penza, Samara, Saratov and Ulyanovsk regions.

The samples from HIV-infected patients with indications of the presence of virological failure of the ART regimen were studied. For the final analysis, the results of the study of samples from 1396 patients were selected. Patients who do not receive ART as well as those lacking amplification in bio-samples were excluded from further analysis.

Informed consent to participate in the study was previously obtained from all patients. The group of interest included 771 males and 625 females with different paths of HIV transmission, aged from 1 to 71 years. The median age was 35 years (male 36 years; female 33 years). A total of 98.9% of patients received ART for more than one year.

Blood plasma was the only material used for investigation. Studies on the identification of mutations associated with HIV DR included the determination of the base sequences of the HIV genome using the ViroSeq™ HIV-1 genotyping system (Celera Diagnostic, Abbott Laboratories, Alameda, CA, USA), and the “AmpliSens^®^ HIV-Resist-Seq” of the Federal State Budgetary Institution of the Central Research Institute of Epidemiology of Rospotrebnadzor (Moscow, Russia) by sequencing of amplified fragments of the gene *pol* (a fragment of the protease gene *pro*, reverse transcriptase *rev*, integrase *int*) of HIV-1, using seven primers included in the kit with the BigDye sequence reaction terminator (Sequencing Module—BigDye v.3.1), on the ABI Prism 3100 genetic analyzer and SeqStudio Applied Biosystems, USA. Sequencing data processing, obtainingn a consensus sequence as well as further analysis were carried out using the analytical software ViroSeq™ HIV-1 genotyping system software v.2.8 (Celera, Alameda, CA, USA) and the Stanford University database (versions 4.3, 5.0, 5.1, 6.0, 6.1, 6.2, 6.3, 7.0, 8.1, 8.2, 8.3, 8.4, 8.5, 8.6, 8.7, 8.8, 8.9; accessed at: http://hivdb.stanford.edu, accessed on 24 August 2022), in order to determine the profile of HIV DR mutations to various antiretroviral drugs.

Of the analyzed sequences, 252 HIV nucleotide sequences were deposited in GenBank, specifically the following: in 2012—37 sequences (JX141197–JX141233); in 2013—35 (KF257850–KF257884); in 2014—35 (KP090065–KP090099); and additionally 70 sequences (KJ722070–KJ722139, together with the Central Research Institute of Epidemiology, Russia), in 2015—15 (KT121451–KT121464); in 2016—15 (KY052009–KY052023); in 2017—15 (MG063795–MG063809); in 2018—15 (MK054185–MK054199); in 2019—15 (MN633414–MN633428). These data are available at the following site: https://www.ncbi.nlm.nih.gov/genbank/release (accessed on 24 August 2022).

The obtained results of the mutation detection were subsequently compared with the data from HIV epidemiological monitoring in the areas of the VFD. The data are available at the following site: https://nniiem.ru/development/informanalit/AIDS.html (accessed on 24 August 2022).

For statistical analysis, the integrated package of the Microsoft Office Excel 2010 application program was employed, using methods of variational statistics, correlation and regression analysis, and calculations of standard deviation errors (m). Statistical significance of differences between the two data samplings were assessed using the Student’s criterion (t) and Fisher’s criterion (F). We considered a two-sided *p*-value below 0.05 to be statistically significant.

## 3. Results

From 2008 to 2019, base sequences were isolated from 1396 blood plasma samples of HIV-infected patients.

A comparative analysis of the results obtained during the periods of 2008–2015 (Group 1) and 2016–2019 (Group 2) was performed. Group 1 consisted of 472 patients (253 male and 219 female; median age, 32 years). Group 2 included 924 patients (518 men and 406 women, median age, 37 years). Patients in Group 2 were older than those in Group 1 (mean age 35.93 ± 0.36 and 29.09 ± 0.56, respectively, *p* < 0.01). The vast majority of them received ART for more than 1 year: Group 1, 97.0%, Group 2, 99.9%. However, the difference between groups was statistically significant (*p* < 0.05). Patients’ characteristics are presented in Table 1.

Of the 472 samples examined between 2008 and 2015, viral strains containing DR mutations were found in 273 (57.8%) samples, and no DR mutations were detected in 199 (42.2%) samples.

The annual indicators of acquired drug resistance (ADR) from 2008 to 2015 were 41.5% (17/41), 55.0% (33/60), 59.3% (51/86), 55.2% (37/67), 57.0% (53/93), 59.1% (13/22), 69.7% (23/33), and 65.7% (46/70), respectively (Table 2).

DR mutations were identified using the algorithm of the Stanford University HIV Drug Resistance Database.

In Group 2 (the period from 2016 to 2019), HIV drug resistance was assessed for 924 samples: 722 (78.1%) HIV strains with DR mutations were found; there was no DR found in 202 (21.9%) samples. From 2016 to 2019, the yearly prevalence of ADR was 62.4% (98/157), 72.4% (147/203), 86.2% (243/282), and 83.0% (234/282), respectively (Table 3).

In Group 1 (2008 to 2015), multiple DR (MDR) was registered in 181 samples (38.3%). MDR suggests that HIV is resistant to antivirals of various pharmacological groups – nucleoside reverse transcriptase inhibitors (NRTIs), non-nucleoside reverse transcriptase inhibitors (NNRTIs), protease inhibitors (PIs), and so on. Mutations to NRTIs + NNRTIs were detected in 151 samples (32.0%); NRTIs + PI in 15 cases; NNRTIs + PI in 1 case; and NRTIs + NNRTIs + PI in 4 samples.

In Group 2 (2016–2019), mutations to two classes of HIV drugs were observed in 553 (59.8%) samples: NRTIs + NNRTIs in 523 cases; NRTIs + PIs in 27 cases; and NNRTIs + PIs in 3 cases. Mutations to three classes of antiretrovirals were detected in 42 HIV base sequences (4.5%).

Generally for Group 1 (2008 to 2015), in the spectrum of DR mutations, mutations associated with NNRTIs (both as single class and as part of MDR) were detected in 48.7% (228 cases). A total of 43.4% (203 cases) were mutations associated with resistance to NNRTIs and 7.9% (37 cases) were mutations to PIs. For Group 2 (2016 to 2019), in the spectrum of DR mutations, mutations of resistance to NRTIs amounted to 47.6% (647 cases), to NNRTIs 46.3% (629), and to PIs 6.1% (83).

The most common mutations that determined resistance in the HIV reverse transcriptase gene and in the HIV protease gene identified in 2008–2019 are presented in Table 4.

During both follow-up periods, the most widespread DR mutations were M184VI (37.9%/61.7%), K65R (2.3%/27.9%), L74V/I (3.8/7.8%), and T215F/Y (7.8%/8.0%), leading to the development of DR to NRTIs, and the replacement of K101E/H/P/Q (15.5%/24.7%), Y181C (3.6%/25.2%), G190S (17.8%/35.3%), and K103N (10.8%/13.5%); contributed to the emergence of resistance to NNRTIs. Mutations that caused high DR in the protease gene were not so common. They were most often detected in 2017 and 2018. In the remaining years viral strains with only single substitutions were registered. The main mutations were M46I (5.7%/4.9%), I50L (2.8%/2.4%), and V82A (0.4%/2.1%), which are causing a high degree of resistance to NFV and ATV, along with SQV.

## 4. Discussion

**Figure 1:**
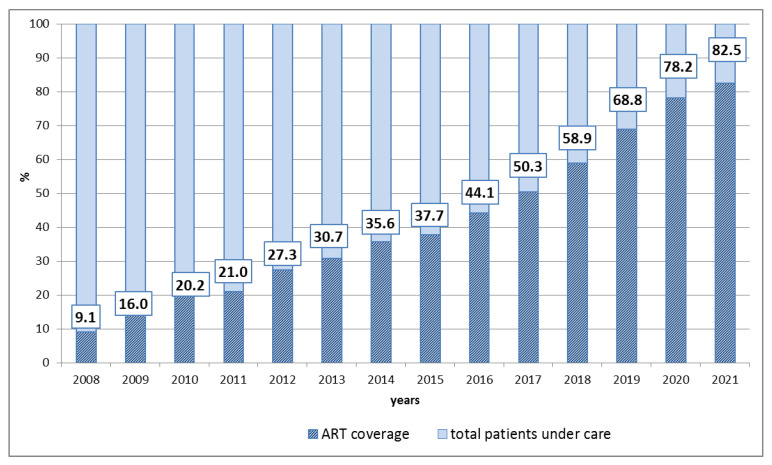
ART coverage of HIV-infected patients under care in the VFD during 2008–2021.

The ART coverage of HIV infected patients is a key factor in determining the development of the epidemic process of HIV infection. The main goals of ART are to increase the lifetime and preserve the quality of life of HIV-infected persons. The systematic use of ART was initiated in Russia in 2006 within the framework of federal state programs. As a result of intensive research and development internationally, eight groups of HIV antiretrovirals with different mechanisms of interference with HIV replication have been developed and approved. In the VFD, the ART coverage of HIV-infected patients increased in 2016 by 4.8-fold, in 2019 by 7.6-fold, and in 2021 by 9.0-fold, compared with 2008 (Figure 1, results are presented in percent of patients).

Initially, WHO recommendations formed the basis for the use of ART in Russia. The WHO Guideline on antiretroviral therapy for HIV infection in adults and adolescents was first published in 2002, and then underwent repeated revisions.

New scientific data appeared regarding the timing of ART initiation, optimal ART regimens, management tactics for HIV co-infections with tuberculosis and chronic viral hepatitis, as well as management approaches in case of treatment failure. At the same time, Russia adjusted its ART protocols and HIV patient management strategies, and changed the list of specific drugs produced and purchased by the country [13,14].

Historically, the WHO recommendations related to prescribing ART and monitoring its effectiveness progressed toward earlier initiation, simplification of therapy regimens, the use of less toxic and more reliable regimens, and to the improvement and simplification of treatment monitoring [15,16].

In 2016, the WHO provided an urgent recommendation to initiate ART for adult people living with HIV (PLWH), regardless of their clinical stage of the disease and their numbers of CD4^+^ cells. The recommendation to ”treat everyone” has led to a large-scale increase in ART coverage in more than 130 countries around the world, including Russia. The availability of treatment monitoring has also increased. In 2017, an increase in the scale of ART was accelerated by the recommendation to initiate therapy quickly, within seven days of the diagnosis of HIV infection; it was also suggested to start therapy on the same day. Patients with advanced stages of HIV disease take priority of the immediate ART start [17].

Russian updated recommendations for the treatment of HIV-infected patients are presented in the clinical recommendations “HIV infection in adults” (2020) [18]. According to this document, ART is currently considered the main component of treatment for patients with HIV infection, which makes it possible to achieve a controlled course of the disease.

In accordance with current regulatory documents in the Russian Federation, if a patient is ready to start ART and has consented, therapy can be initiated immediately, just after diagnosis of HIV infection is established, and so long as there are no clinical contraindications for taking HIV drugs.

At the same time, early initiation of therapy also carries potential risks that arise from the long-term use of antiretrovirals, such as late toxic effects and the development of DR. The key points in ensuring the effectiveness of therapy are the continuous availability of medicines as well as high adherence of patients to the prescribed regimens. In order to optimize patients’ adherence to ART, it is of great importance to prevent the development of side effects, to recognize the potentially serious complications, and to employ new HIV drugs that have more favorable effects on the body in addition to a higher barrier to resistance [19,20].

Drug resistance can be divided into acquired drug resistance (ADR), which is detected in patients who have previously received treatment, and transmissible drug resistance (TDR) in patients who are ART naïve. Ignoring TDR can lead to ineffective treatment with antiretroviral regimens, and ADR is often associated with virological failure and can increase the burden of treatment [21,22].

While TDR is well documented, fewer studies have reported the prevalence of ADR among patients on long-term therapy [23]. A systematic review that was conducted under conditions of limited resources reported a steady increase in the frequency of HIV DR mutations with prolonged use of antiretrovirals [24]. The M184V mutation to NRTIs, which causes high resistance to 3TC, is most often detected. The next most common ADR mutation is K103N of the NNRTIs group, which causes high-level resistance to NVP and variable resistance to EFV [25].

In our study, it was found that over the eight years from 2008 to 2015, the proportion of patients with identified mutations of DR to both one and several classes of HIV medicines increased by more than 1.5-fold. Significant increases compared to previous years were noted in 2009 (1.3-fold) and in 2014 (1.2-fold), whereas in 2009–2013, as well as in 2014–2015, the annual frequency of DR registration was recorded at approximately the same level. In general, 57.8% patients in Group 1 had DR.

During the period of 2016–2019, the frequency of detection of DR mutations increased at a faster rate compared to the previous period. During the four years of follow-up, the frequency of occurrence of HIV strains with DR mutations increased 1.9-fold. Significant increases compared to previous years were recorded in 2017 and in 2018 (1.2 times annually). In 2019, compared to 2018, the proportion of patients with DR mutations was slightly lower. In general, during the analyzed period, HIV DR was detected in 78.1% of the examined patients, which is statistically more often than in the previous period (*p* < 0.01).

Cases of MDR were registered in both groups of patients (38.3% and 64.4%, respectively). The proportion of such patients was statistically higher in Group 2 (*p* < 0.01). In general, during the follow-up period, an increase in the proportion of patients with MDR was detected from 17.1% in 2008 up to 72.7% in 2019.

**Figure 2:**
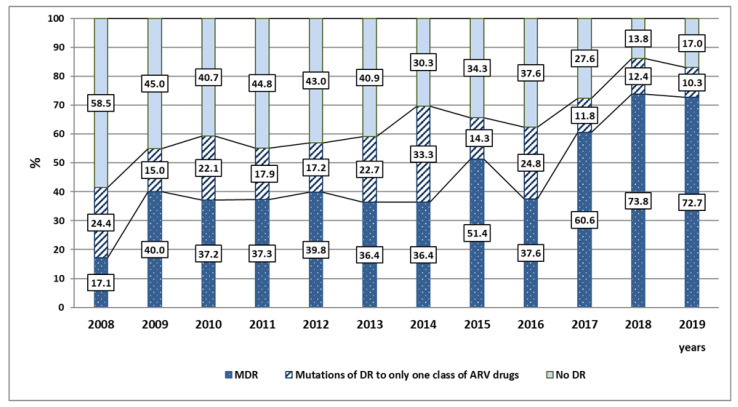
Dynamics of the structure of HIV DR in patients from the VFD during 2008–2019.

Similarly with the dynamics of the increase in the prevalence of DR in general, significant increases in the proportion of MDR cases compared to the previous year were noted in the first analyzed period in 2009 and 2014 (2.3 and 1.4 times, respectively), and in the second period in 2017 and 2018 (1.6 and 1.2 times, respectively) (Figure 2, results are presented in percent of patients).

**Figure 3:**
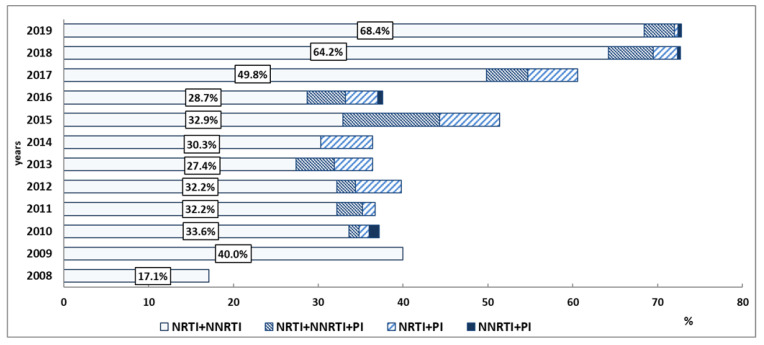
The structure of HIV MDR among ART-experienced patients from the VFD in 2008–2019.

A combination of resistance to two classes of drugs, nucleoside and non-nucleoside reverse transcriptase inhibitors, prevails in the structure of multiple DR in both periods of observation. The share of resistance to NRTIs + NNRTIs increased 1.9-fold from 2008 to 2015, with an even faster increase in the share of this variant of DR noted from 2016 to 2019 (2.3-fold). When comparing the two analyzed periods, the proportion of such patients increased 1.8-fold. DR to NRTIs after 2016 began to be registered more often as part of MDR than to a single class of antiretrovirals. In accordance with up-to-date clinical recommendations “HIV infection in adults”, medicines of these pharmacological groups are included in the preferred first-line ART regimens, including two NRTIs and a third drug, which may belong to NNRTIs, PIs or PI/r, or INSTIs [26,27,28,29]. In the period from 2016 to 2019, DR to three classes of HIV drugs (NRTI + NNRTI + PI) remained at an average level of 4.5%. The smallest proportion of MDR is accounted for by the combination of NNRTI + PI, which was detected in four patients, in total, in 2010, 2016, 2018, and 2019 (Figure 3, results are presented in percent of patients).

Since 2016, there has been an expansion in the spectrum of dominant mutations related to DR in HIV strains circulating in the VFD.

The main mutation that caused a high level of resistance to NRTIs during 2016–2019 continued to be M184V/I, averaging 60.8% in the district. The prevalence of this mutation in the specified period was 1.9 times higher than that in the period up to 2016.

**Figure 4:**
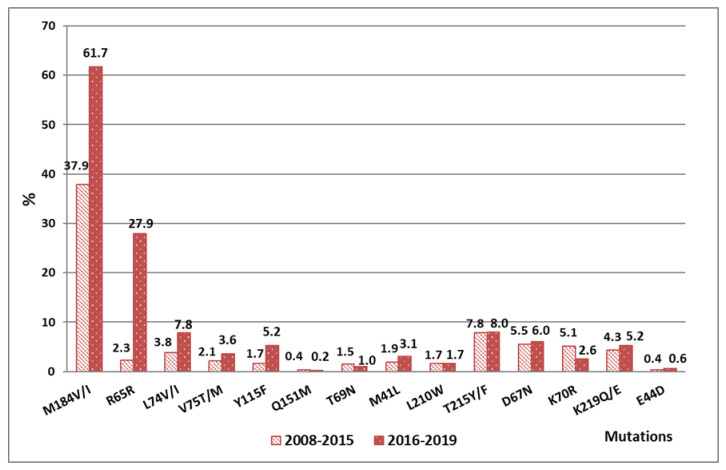
The prevalence of different mutations to NRTIs in the VFD in 2008–2015 and 2016–2019.

The M184V/I mutation overcomes the effects of 3TC and FTC, and reduces the sensitivity of the virus to them by more than 100 times; it also causes low-level resistance to ddI and ABC. Frequent occurrence of this mutation is expected, and is a consequence of the use of 3TC and FTC as part of first-line ART regimens in Russia. This substitution does not cause cross-resistance to other NRTIs. Its presence, on the contrary, increases the sensitivity of the virus to TDF, d4T, and AZT, and is associated with a clinically significant decrease in HIV replication [26,27,28]. In Western Europe, the prevalence of this mutation is lower compared to Asia, Africa, and Latin America [29,30]. This can be explained by differences in HIV treatment regimens, as 3TC and FTC are used less frequently in these regions (Figure 4, results are presented in percent of patients).

In the group of mutations to NRTIs, there is a significant increase in the frequency of occurrence of K65R replacement to an average of 27.9% during the period from 2016 to 2019, which is associated with more frequent use of TDF instead of AZT and d4T as a part of first-line therapy. From 2008 to 2015, its contribution to the maintenance of virus resistance was insignificant, on average 2.3%. K65R is a characteristic mutation associated with a 3–4-fold increase in virus resistance to TDF. It also causes medium-level resistance to ddI and ABC; low-level resistance to d4T; and is associated with hypersensitivity to AZT [4,31,32,33].

The frequency of occurrence of the group of DR mutations to thymidine analogues (TAMs)—M41L, L210W, T215F/Y, D67N, K70R, K219Q/E, and E44D—was maintained at the same level during both periods of observation, and averaged 26.7% and 27.3%, respectively. These mutations cause high and medium HIV resistance to AZT and d4T, and low-level resistance in response to a regimen containing ABC, ddI, or TDF.

During the period of 2016–2019, no mutations of Q151M and T69N were detected. During the period of 2008–2015, these mutations occurred in nine patients.

Compared with the period up to 2016, the frequencies of registration of mutations L74V/I, V75T/M, and Y115F/Y increased by an average of 1.9 times. The L74V mutation was most often detected in patients receiving ABC or ddI. This mutation leads to a decrease in virus sensitivity to ABC by more than five times and to ddI by two times, and increases the susceptibility of HIV to AZT. Mutation Y115F/Y causes DR to ABC and TDF [33,34,35].

**Figure 5:**
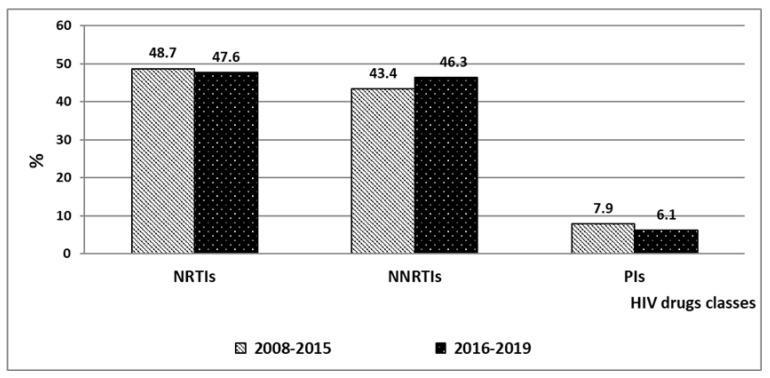
The frequencies of detection of DR mutations to various classes of antiretrovirals in the VFD from 2008 to 2015 and from 2016 to 2019.

A feature of antiretrovirals from the NNRTI class is that they contribute to the rapid emergence of drug-resistant HIV variants. NNRTIs have a low genetic barrier, and bear a high risk of developing cross-resistance. The presence of at least one of the mutations G190A/S, K103N, Y181C, or K101E/H/P in the active center of the reverse transcriptase enzyme prevents the binding of several drugs at once, rendering them ineffective. After 2016, drugs of the NNRTI group were more often used in first-line ART regimens than in the previous period. In the period 2008–2015, the incidence of DR to NNRTIs (43.4%) in the spectrum of mutations registered in the VFD was less than the DR to NRTIs (48.7%, difference is statistically insignificant, *p* > 0.05). In the period from 2016 to 2019, the proportion of cases of DR to NNRTIs (both as single class and as part of MDR) was up to 46.3%, but still ranked below the DR to NRTIs (47.6%) in the total number of cases of drug resistance mutations. However, this frequency increase was not statistically significant (*p* > 0.05). We also found that the spread of NRTI and NNRTI resistance mutations both before and after 2016 has only slightly changed (*p* > 0.05) (Figure 5, results are presented in percent of DR mutations’ number). At the same time, the frequencies of occurrence of DR mutations to PIs in both periods were significantly lower compared with DR mutations to NRTIs and NNRTIs alike (*p* < 0.05).

The main mutation that caused a high level of DR to NNRTIs both in Group 1 (2008–2015) and in Group 2 (2016–2019) was the replacement of G190S (17.9%, 35.3%, respectively). At the same time, the frequency of its occurrence increased by a factor of two after 2016. The G190S mutation causes a high level of resistance to NVP and EFV, and is most often registered in HIV-1 strains of subtype A6 dominating in Russia [10,33,36].

**Figure 6:**
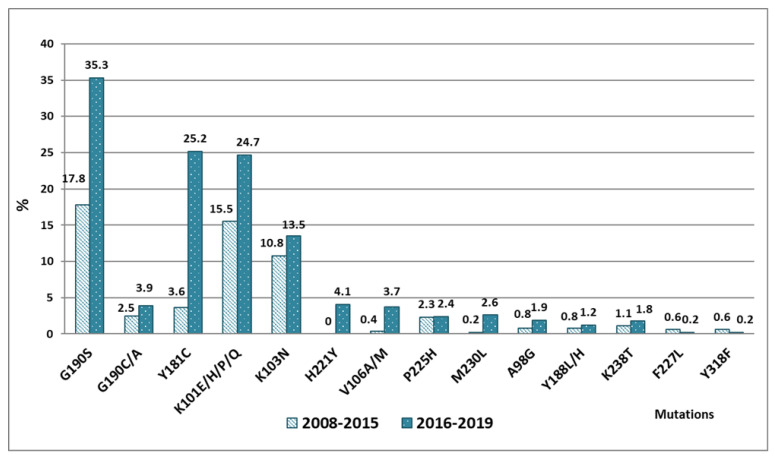
The prevalence of different mutations to NNRTIs in the VFD during 2008–2015 and 2016–2019.

During the period from 2016 to 2019, the occurrence of mutations Y181C (3.6%, 25.2%) increased 7-fold and K101E/H/P (15.5%, 24.7%) increased 1.6-fold compared to previous years, replacing K103N from the dominant position. The prevalence of the K103N mutation, which fundamentally reduces the sensitivity of the virus to NVP and EFV (about 50-fold and 20-fold, respectively), changed slightly during the observation periods, and comprised 10.8% and 13.5%, respectively. The Y181C mutation causes more than a 50-fold decrease in the susceptibility of the virus to NVP, and a 10–15-fold decrease in susceptibility to ETR and RPV. The K101E/H/P mutation causes resistance to all drugs of the NNRTI group (NVP, ETR, EFV, and RPV), and its prevalence continues to increase from year to year. The mutations G190C/A, V106A/M, A98G, M230L, Y188L/H, K238T, and H221Y were recorded in the virus genome throughout all the years of the study, and determined high-level resistance to NVP and EFV and ETR (Figure 6, results are presented in percent of patients). The data obtained are consistent with similar studies from low- and middle-income countries [37].

**Figure 7:**
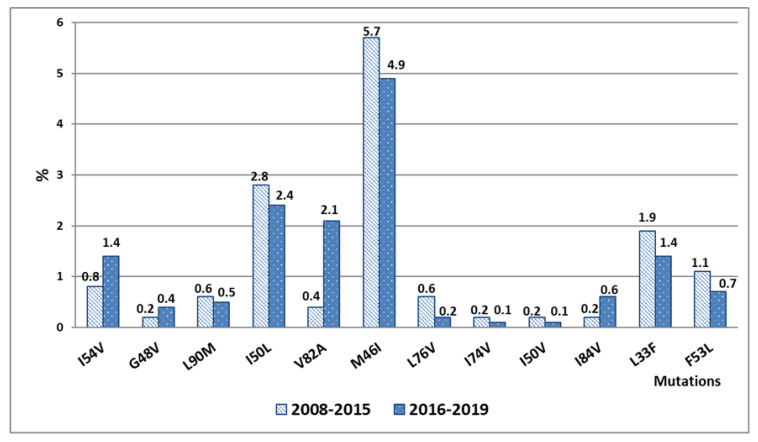
The prevalence of different mutations to PIs in the VFD during 2008–2015 and 2016–2019.

During the period from 2016 to 2019, some decrease in the frequency of DR to PI in the spectrum of mutations was observed. It comprised 6.1% compared to 7.9% in 2008–2015 (difference is statistically insignificant, *p* > 0.05). The low level of resistance to PI can be explained by a high genetic barrier to resistance, which determines a lower probability of developing resistance. Mutations M46I (5.7%, 4.9%), I50L (2.8%, 2.4%), and V82A (0.4%, 2.1%) were detected most frequently in all years of observation. The M46I mutation causes a decrease in the sensitivity of the virus to NFV, IDV, FPV, ATV, and LPV. The second most common mutation is I50V, which causes a high degree of resistance to ATV. Replacement of V82A reduces susceptibility to IDV and LPV and causes cross-resistance to ATV and NFV [10,33,38,39]. The remaining mutations were determined in single cases and not in every year (Figure 7, results are presented in percent of patients).

Currently in the VFD, INSTIs have become widely included in the treatment regimens for HIV-infected patients. This pharmacological group of antiretrovirals meets the basic requirements for ensuring conditions of patients’ adherence to therapy: single dosing, safety, absence of side effects, and good tolerability [8,40]. In accordance with clinical guidelines for the management of adult patients with HIV infection adopted in Russia, DGV is included in preferred first-line ART regimens (TDF + 3TC + DTG; TDF + FTC + DTG), as well as in parts of alternative schemes (ABC + 3TC + DTG) [29]. If it is impossible to employ preferred and alternative regimens, special case regimens are prescribed in which INSTI is included as a third drug: RAL or BIC or EVG as an alternative to EFV or DTG. BIC and EVG are available as ingredients for medicines with a fixed combination of doses (Genvoya® and Biktarvy®), in order to increase adherence to therapy and convenience of drug administration.

**Figure 8:**
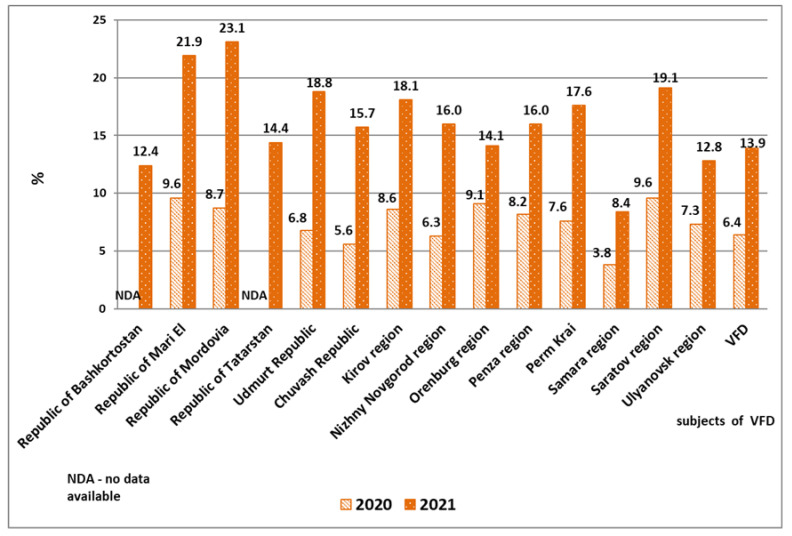
Coverage of HIV-positive patients from the VFD with INSTI-containing ART regimens in 2020–2021.

INSTI-based ART regimens have become widely employed in the VFD in recent years. In the process of epidemiological monitoring of HIV DR, the frequency of INSTI administration in subjects of the VFD in 2020–2021 was analyzed. In 2021, an average of 13.9% of HIV-infected patients in the district received an ART regimen that included INSTIs, which was 1.9-fold higher than the usage of such regimens in 2020 (6.4%). This trend was observed both in areas with a high prevalence of HIV infection among the population (Perm Krai, Samara, Orenburg, and Ulyanovsk regions) as well as in less affected areas. The largest percentage of patients receiving INSTIs in 2021 was observed in the republics of Mordovia and Mari El, as well as in the Saratov region. The minimum coverage of patients with therapy containing INSTIs was registered in the Samara and Ulyanovsk regions, and in the Republic of Bashkortostan (Figure 8, results are presented in percent of patients).

Currently, in almost all areas in the district, patients receive an INSTI-containing regimens for more than 6 months. On average in the VFD, in 2021 the percentage of such patients was 78.5; in 2020 it was 65.3 (*p* < 0.01). The largest proportions of patients receiving INSTIs for more than 6 months are registered in the Kirov, Nizhny Novgorod, and Samara regions (91.6%, 93.5%, and 94.6%, respectively). In the Penza region, only 17.3% of HIV-infected people received INSTI-based ART regimens for more than 6 months.

**Figure 9:**
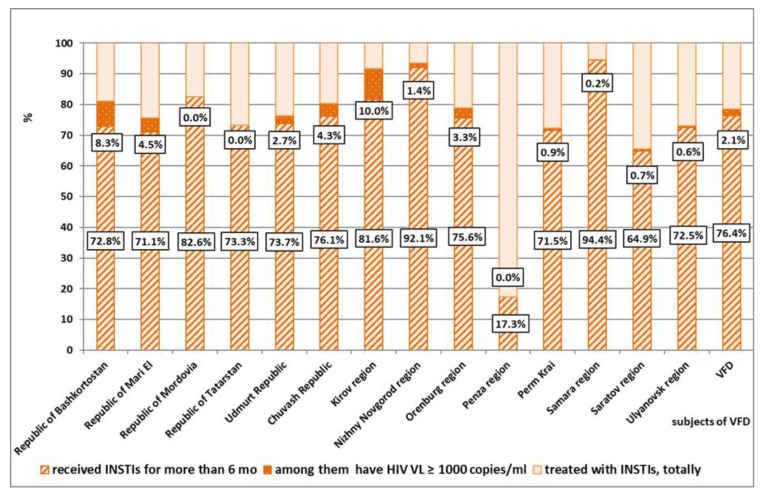
Proportions of patients receiving INSTI-based ART regimens in the different VFD areas (as of 1 September 2021).

Integration inhibitors have a consistently high antiviral effect, resulting in maintenance of HIV VL at an undetectable level for a long time. An analysis of the effectiveness of INSTI-containing regimens showed that the percentage of patients who, after 6 months of treatment, had an HIV VL greater than 1000 copies/mL decreased 1.9-fold in 2021 compared to 2020, which amounted to 2.1%; this confirms a good INSTI efficacy profile (Figure 9, results are presented in percent of patients).

In the laboratory of Privolzhsky Okrug Center for the Prevention and Control of AIDS in 2021, for the first time, studies of DR mutations of the HIV-1 genome to INSTI were conducted for four areas of the VFD: the Nizhny Novgorod and Ulyanovsk regions, the Republic of Mordovia and the Chuvash Republic. The study was conducted in 23 HIV-infected patients who received INSTI-based treatment regimens, both at the time of the study and earlier (several years ago). No DR mutations to INSTIs were detected in 16 samples.

Five types of relevant DR mutations to INSTI were identified in seven samples. A secondary (associated) T97A mutation was detected in three samples, which in combination with primary mutations, reduces the virus sensitivity to all INSTIs. Primary mutations Y143R and Q148R were also identified in two samples, G140A/S in three samples, and E138K in one sample. All of these mutations cause a high level of resistance to first-generation INSTIs.

In 20 of the samples studied, including those in which resistance to INSTIs was not detected, a mutation of polymorphism L74I was observed, specific for the A6 subtype of HIV, which is dominant in the Russian Federation.

A distinctive feature of INSTIs is their high barrier to resistance. In order to reduce the effectiveness of drugs, a virus must carry combinations of certain mutations: primary, secondary, and mutations of polymorphism. Different combinations of these mutations cause resistance to various INSTIs. In three patients with known resistance to INSTIs, multiple changes in the ART regimen had taken place, where RAL and DGV were replaced by one another several times in a row, resulting in the development of DR. The only treatment regimen containing RAL was ineffective in four people.

In our study, high and moderate DR to first-generation INSTIs was detected with a combination of the following types of mutations:

− one primary and one mutation of polymorphism (Y143R + L74I and G140S + L74I), in two strains;
− one primary, one secondary, and one mutation of polymorphism(Y143R + T97A + L74I), in one strain.

The combination of two primary mutations, G140A + Q148R, causes moderate DR to DGV and BIC in one strain.

The combination of three primary mutations, E138K, G140A, and Q148R, and mutations of polymorphism L74I identified in one sample, leads to the development of high DR to all INSTIs of both the first and second generations.

The combination of one secondary mutation and one mutation of polymorphism, T97A + L74I, which we discovered in two strains, determines the initial degree of DR to first-generation INSTIs.

## 5. Conclusions

The performed analysis of prevalence and structure of HIV DR mutations to antiretrovirals makes it possible, within the framework of epidemiological surveillance, to develop approaches to prevent the occurrence as well as limit the spread of resistant HIV strains in the VFD. The results of this study are employed in the practical research of specialists at the Centers for the Prevention and Control of AIDS and Infectious Diseases in the VFD areas, who aim to determine the specific cause of the virological inefficiency of ART and to select an alternative treatment regimen in consideration of the data from virus genotyping.

Since 2016, a 1.9-fold increase in the frequency of registration of HIV strains with DR mutations has been revealed. The proportion of HIV strains with MDR to two classes of drugs, NRTIs and NNRTIs, is increasing. In 2016–2019, the proportion of patients with resistance to NRTIs + NNRTIs increased by 1.8 times compared to the period before 2016.

An expansion of the spectrum of dominant DR mutations K65R, TAMs, Y181C, and K101ER was revealed, which led to the circulation in the VFD of HIV strains that were resistant to most drugs of the NRTI and NNRTI classes.

The frequency of registration of DR mutations to the PI pharmacological group in all years of observation was low. The decrease in the frequency of PI DR mutations in 2016–2019 compared to the period up to 2016 is statistically insignificant.

In the VFD, the proportion of HIV-infected people receiving INSTI-based treatment regimens has showed a two-fold increase over the period from 2020 to 2021, and comprised on average 14.0% of the number of ART-experienced patients. ADR to INSTIs was revealed in 7 out of 23 samples from HIV-infected patients with various combinations of mutations (E138K, G140A, Q148R, Y143R, T97A, and L74I).

HIV integration inhibitors meet the basic requirements that are necessary to enable patients to be adherent to therapy. However, we have noted that repeated replacement of INSTIs in ART regimens leads to the emergence and accumulation of DR mutations even to such effective drugs. The scale-up of INSTI deployment in ART regimens determines the relevance of further monitoring that is required for both ADR and TDR of HIV to this class of drugs.

## Figures and Tables

**Table 1:** Baseline characteristics of ART-experienced HIV-positive patients with virological failure in the VFD in 2008–2015 (Group 1) and 2016–2019 (Group 2).

Characteristics	Total (*n* = 1396)	Group 1 (*n* = 472)	Group 2 (*n* = 924)
Median (IQR) age, years	35 (1–71)	32 (1–57)	37 (1–71)
<15 years	140 (10.0%)	77 (16.3%)	63 (6.8%)
15–44 years	1070 (76.6%)	362 (76.7%)	708 (76.6%)
45–60 years	178 (12.8%)	33 (7.0%)	145 (15.7%)
>60 years	8 (0.6%)	0	8 (0.9%)
Sex			
male	771 (55.2%)	253 (53.6%)	518 (56.1%)
female	625 (44.8%)	219 (46.4%)	406 (43.9%)
Stage of HIV disease			
2	11 (0.8%)	4 (0.8%)	7 (0.8%)
3	143 (10.2%)	59 (12.5%)	84 (9.1%)
4	996 (71.3%)	350 (74.2%)	646 (69.9%)
4A	465 (33.3%)	221 (46.8%)	244 (26.4%)
4Б	287 (20.5%)	90 (19.1%)	197 (21.3%)
4B	244 (17.5%)	39 (8.3%)	205 (22.2%)
No data available	246 (17.6%)	59 (12.5%)	187 (20.2%)
Time from ART onset			
<1 year	15 (1.1%)	14 (3.0%)	1 (0.1%)
1 year and more	1381 (98.9%)	458 (97.0%)	923 (99.9%)

**Table 2:** The occurrence of HIV drug resistance (DR) in ART-experienced patients in the VFD in 2008–2015.

Years	2008	2009	2010	2011	2012	2013	2014	2015	Total
Number of samples with received base sequence	41	60	86	67	93	22	33	70	472
Number of samples with mutations of DR	17 (41.5%)	33 (55.0%)	51 (59.3%)	37 (55.2%)	53 (57.0%)	13 (59.1%)	23 (69.7%)	46 (65.7%)	273 (57.8%)
Number of samples without mutations of DR	24 (58.5%)	27 (45.0%)	35 (40.7%)	30 (44.8%)	40 (43.0%)	9 (40.9%)	10 (30.3%)	24 (34.3%)	199 (42.2%)
DR to one class of HIV drugs	10 (24.4%)	9 (15.0%)	19 (22.1%)	12 (17.9%)	16 (17.2%)	5 (22.7%)	11 (33.3%)	10 (14.3%)	92 (19.5%)
NRTI *	6 (14.6%)	6 (10.0%)	10 (11.6%)	2 (3.0%)	10 (10.7%)	2 (9.1%)	6 (18.2%)	6 (8.6%)	48 (10.2%)
NNRTI **	2 (4.9%)	2 (3.3%)	8 (9.3%)	10 (14.9%)	6 (6.5%)	2 (9.1%)	4 (12.1%)	3 (4.3%)	37 (7.8%)
PI ***	2 (4.9%)	1 (1.7%)	1 (1.2%)	0	0	1 (4.5%)	1 (3.0%)	1 (1.4%)	7 (1.5%)
MDR ****	7 (17.1%)	24 (40.0%)	32 (37.2%)	25 (37.3%)	37 (39.8%)	8 (36.4%)	12 (36.4%)	36 (51.4%)	181 (38.3%)
NRTI/NNRTI	7 (17.1%)	24 (40.0%)	29 (33.6%)	22 (32.8%)	30 (32.2%)	6 (27.4%)	10 (30.3%)	23 (32.9%)	151 (32.0%)
NRTI/PI	0	0	1 (1.2%)	1 (1.5%)	5 (5.4%)	1 (4.5%)	2 (6.1%)	5 (7.1%)	15 (3.2%)
NNRTI/PI	0	0	1 (1.2%)	0	0	0	0	0	1 (0.2%)
NRTI/NNRTI/PI	0	0	1 (1.2%)	2 (3.0%)	2 (2.2%)	1 (4.5%)	0	8 (11.4%)	14 (2.9%)

* NRTI—nucleoside reverse transcriptase inhibitors; ** NNRTI—non-nucleoside reverse transcriptase inhibitors (NNRTIs); *** PI—protease inhibitors; **** MDR—multiple DR.

**Table 3:** The occurrence of HIV DR in ART-experienced patients in the VFD in 2016–2019.

Years	2016	2017	2018	2019	Total
Number of samples with received base sequence	157	203	282	282	924
Number of samples with mutations of DR	98 (62.4%)	147 (72.4%)	243 (86.2%)	234 (83.0%)	722 (78.1%)
Number of samples without mutations of DR	59 (37.6%)	56 (27.6%)	39 (13.8%)	48 (17.0%)	202 (21.9%)
DR to one class of HIV drugs	39 (24.8%)	24 (11.8%)	35 (12.4%)	29 (10.3%)	127 (13.7%)
NRTI	18 (11.5%)	17 (8.4%)	16 (5.7%)	4 (1.4%)	55 (6.0%)
NNRTI	17 (10.8%)	5 (2.5%)	18 (6.4%)	21 (7.4%)	61 (6.6%)
PI	4 (2.5%)	2 (0.9%)	1 (0.4%)	4 (1.4%)	11 (1.2%)
MDR	59 (37.6%)	123 (60.6%)	208 (73.8%)	205 (72.7%)	595 (64.4%)
NRTI/NNRTI	45 (28.7%)	101 (49.8%)	184 (65.2%)	193 (68.4%)	523 (56.6%)
NRTI/PI	6 (3.8%)	12 (5.9%)	8 (2.8%)	1 (0.4%)	27 (2.9%)
NNRTI/PI	1 (0.6%)	0	1 (0.4%)	1 (0.4%)	3 (0.3%)
NRTI/NNRTI/PI	7 (4.5%)	10 (4.9%)	15 (5.3%)	10 (3.6%)	42 (4.5%)

**Table 4:** Frequencies of mutations causing a high degree of resistance in reverse transcriptase and protease genes of HIV in the VFD during 2016–2019.

Years	2008	2009	2010	2011	2012	2013	2014	2015	TOTAL	2016	2017	2018	2019	TOTAL
Mutations	Number of Samples with Received Base Sequence													
	41	60	86	67	93	22	33	70	472	157	203	282	282	924
**NRTIs**														
M184V/I	11	21	29	21	50	7	20	20	179 (37.9%)	92	109	188	181	570 (61.7%)
K65R	1	5	2	2	2	0	0	1	13 (2.3%)	3	27	108	120	258 (27.9%)
L74V/I	0	1	1	1	2	1	5	7	18 (3.8%)	17	18	15	22	72 (7.8%)
V75T/M	2	0	0	3	2	0	0	3	10 (2.1%)	6	5	14	8	33 (3.6%)
Y115F/Y	0	0	0	2	2	0	0	4	8 (1.7%)	11	8	14	15	48 (5.2%)
Q151M	0	1	0	1	0	0	0	0	2 (0.4%)	1	1	0	0	2 (0.2%)
T69N	2	0	0	0	1	0	1	3	7 (1.5%)	2	6	1	0	9 (1.0%)
M41L	0	1	0	0	5	0	1	2	9 (1.9%)	9	9	8	3	29 (3.1%)
L210W	0	0	2	0	3	1	0	2	8 (1.7%)	4	6	4	2	16 (1.7%)
T215F/Y	2	2	7	4	10	5	2	5	37 (7.8%)	17	22	24	11	74 (8.0%)
D67N	1	0	4	4	5	2	3	7	26 (5.5%)	6	10	20	19	55 (6.0%)
K70R	1	2	2	3	5	2	3	6	24 (5.1%)	1	2	14	7	24 (2.6%)
K219Q/E	2	2	1	4	1	1	5	4	20 (4.3%)	5	11	13	19	48 (5.2%)
E44D	0	0	0	0	0	0	1	1	2 (0.4%)	0	2	4	0	6 (0.6%)
**NNRTIs**														
K101E/H/P/Q	5	12	12	8	14	2	7	13	73 (15.5%)	28	49	75	76	228 (24.7%)
Y181C	2	1	4	2	2	0	1	5	17 (3.6%)	12	24	105	92	233 (25.2%)
G190S	4	10	11	11	21	4	6	17	84 (17.8%)	27	60	121	118	326 (35.3%)
G190C/A	1	3	2	0	4	1	0	0	12 (2.5%)	4	4	15	13	36 (3.9%)
V179D/E/T	1	0	1	2	3	0	0	2	9 (1.9%)	17	19	22	14	72 (7.8%)
E138A	0	0	0	0	0	0	0	0	0	13	20	24	30	87 (9.4%)
V108I	0	0	0	3	1	0	2	1	7 (1.5%)	3	1	4	6	14 (1.5%)
K103N	0	3	9	14	10	3	2	10	51 (10.8%)	19	19	51	36	125 (13.5%)
P225H	0	1	5	0	0	0	1	4	11 (2.3%)	4	6	6	6	22 (2.4%)
H221Y	0	0	0	0	0	0	0	0	0	1	10	14	13	38 (4.1%)
V106A/M	0	0	0	0	1	1	0	0	2 (0.4%)	6	7	4	17	34 (3.7%)
A98G	1	0	1	1	2	0	0	0	4 (0.8%)	1	3	8	6	18 (1.9%)
M230L	0	0	0	0	0	0	0	1	1 (0.2%)	0	4	12	8	24 (2.6%)
Y188L/H	1	1	0	0	0	0	1	1	4 (0.8%)	2	0	3	6	11 (1.2%)
K238T	0	0	4	0	1	0	0	0	5 (1.1%)	7	6	2	2	17 (1.8%)
F227L	0	0	0	0	1	1	1	0	3 (0.6%)	0	0	0	2	2 (0.2%)
**PIs**														
I54V	0	0	0	1	1	1	0	1	4 (0.8%)	1	5	6	1	13 (1.4%)
G48V	0	0	0	0	1	0	0	0	1 (0.2%)	0	0	3	1	4 (0.4%)
L90M	1	0	0	0	1	0	0	1	3 (0.6%)	1	2	1	1	5 (0.5%)
I50L	0	0	1	2	3	0	2	5	13 (2.8%)	3	9	5	5	22 (2.4%)
V82A	0	0	0	1	0	0	0	1	2 (0.4%)	5	8	4	2	19 (2.1%)
M46I	10	0	3	1	3	2	2	6	27 (5.7%)	9	12	13	11	45 (4.9%)
L76V	0	0	0	1	1	0	0	1	3 (0.6%)	0	0	1	1	2 (0.2%)
I74V	0	0	0	0	0	0	0	1	1 (0.2%)	0	0	0	1	1 (0.1%)
I50V	0	0	0	0	0	0	0	1	1 (0.2%)	0	0	0	1	1 (0.1%)
I84V	0	0	0	0	0	1	0	0	1 (0.2%)	2	1	2	1	6 (0.6%)
L33F	1	0	0	1	1	0	2	4	9 (1.9%)	6	0	0	7	13 (1.4%)
F53L	0	0	1	0	1	1	0	2	5 (1.1%)	2	2	1	2	7 (0.7%)

## Data Availability

Publicly available data sets were analyzed in this study. This data can be found at the following site: http://epid-atlas.nniiem.ru/a03_data_main_aid.html#, accessed on 24 August 2022 Extra data included in this study are available upon request by contacting the corresponding author.

## Abbreviations

ABC	Abacavir
ATV	Atazanavir
AZT	Zidovudine
BIC	Bictegravir
DGV	Dolutegravir
DRV	Darunavir
ddI	Didanosine
d4T	Stavudine
EFV	Efavirenz
ETR	Etravirine
EVG	Elvitegravir
FPV	Fosamprenavir
FTC	Emtricitabine
IDV	Indinavir
LPV	Lopinavir
LPV/r	Kaletra
NFV	Nelfinavir
NVP	Nevirapine
RAL	Raltegravir
RPV	Rilpivirine
SQV	Saquinavir
TDF	Tenofovir
3TC	Lamivudine
